# Supplementary material for: Patient satisfaction in outdoor department of primary health care facilities in Rohingya refugee camps in Bangladesh: A cross-sectional study
Source: PLoS One. 2026 Jan 13;21(1):e0336811. doi: 10.1371/journal.pone.0336811 (PMC12798992; doi:10.1371/journal.pone.0336811)
Supplement: S1 File — (PDF) [file pone.0336811.s001.pdf]

Scoring system of Patient Satisfaction Questionnaire (PSQ)-18

Table A. Questions Items of PSQ-18

Item No.	Questions of PSQ-18
1	Doctors are good about explaining the reason for medical tests
2	I think my doctor's office has everything needed to provide complete medical care
3	The medical care I have been receiving is just about perfect
4	Sometimes doctors make me wonder if their diagnosis is correct
5	I feel confident that I can get the medical care I need without being set back financially
6	When I go for medical care, they are careful to check everything when treating and examining me
7	I have to pay for more of my medical care than I can afford
8	I have easy access to the medical specialists I need
9	Where I get medical care, people have to wait too long for emergency treatment
10	Doctors act too businesslike and impersonal toward me
11	My doctors treat me in a very friendly and courteous manner
12	Those who provide my medical care sometimes hurry too much when they treat me
13	Doctors sometimes ignore what I tell them
14	I have some doubts about the ability of the doctors who treat me
15	Doctors usually spend plenty of time with me
16	I find it hard to get an appointment for medical care right away
17	I am dissatisfied with some things about the medical care I receive
18	I am able to get medical care whenever I need it

Table B. Scoring Items for PSQ-18

Question Item Numbers	Original Response	Scored Value
1, 2, 3, 5, 6, 8, 11, 15, 18	Strongly Agree ---------->	5
	Agree ---------->	4
	Uncertain ---------->	3
	Disagree ---------->	2
	Strongly Disagree ---------->	1
4, 7, 9, 10, 12, 13, 14, 16, 17	Strongly Agree ---------->	1
	Agree ---------->	2
	Uncertain ---------->	3
	Disagree ---------->	4
	Strongly Disagree ------->	5

Table C. Creating Scale Scores for PSQ-18

**Scale**	**Average These Items**
General Satisfaction	3, 17
Technical Quality	2, 4, 6, 14
Interpersonal Manner	10, 11
Communication	1, 13
Financial Aspects	5, 7
Time spent with Doctor	12, 15
Accessibility and Convenience	8, 9, 16, 18
Overall Satisfaction	1-18

Note: Items within each scale are averaged after scoring as shown in Table B
