# Supplementary material for: Patient satisfaction in outdoor department of primary health care facilities in Rohingya refugee camps in Bangladesh: A cross-sectional study
Source: PLoS One. 2026 Jan 13;21(1):e0336811. doi: 10.1371/journal.pone.0336811 (PMC12798992; doi:10.1371/journal.pone.0336811)
Supplement: S2 File — (PDF) [file pone.0336811.s002.pdf]

Study Protocol

Patient satisfaction in outdoor department of primary health care facilities in Rohingya refugee camps in Bangladesh: A cross-sectional study

EXECUTIVE SUMMARY

Adoption of patient-centric strategy in modern healthcare system is considered as a significant public health intervention which has reinforced the rights of patients as clients on top of getting treated from illness. Periodic assessment on satisfaction of patients is a recommended activity to generate policy recommendations for decision makers and associated partners to achieve the standardized satisfaction level of healthcare consumers. Ownership in implementation of treatment plan, adherence to medication schedules and commitment to self-care of own health are closely related to level of patient satisfaction. For developing countries like Bangladesh, which is already burdened with nearly one million Rohingya population displaced from Myanmar, examining the patient satisfaction status is an important exercise for strengthening health sector strategies in a people-focused approach.

The objective of this study is to assess the level of patient satisfaction among beneficiaries taking outpatient healthcare services from health facilities in Rohingya camps. Difference in status of patient satisfaction across sociodemographic and health related variables with will also be explored in this research. Required data will be collected from five randomly selected primary health care facilities of Rohingya camps in Cox's Bazar district of Bangladesh. Following the systematic random sampling method, a total of 810 beneficiaries will be interviewed by trained field staff.

This study will provide valuable information on distribution of study participants across different independent variables as well as different aspects of patient satisfaction. Data will also be generated on relationship of general satisfaction of patients with different sociodemographic and health variables.

The findings of this study will help policy makers to modify delivery of essential health service package in health facilities of Rohingya camp settings tailored to the needs and expectations of healthcare beneficiaries.

TABLE OF CONTENTS

**Contents**	**Page No.**
Title Page	1
Executive Summary	2
Table of Content	3-4
Abbreviations	5
**CHAPTER I:** INTRODUCTION	6-11
1.1 Introduction	6-7
1.2 Justification of the Study	8
1.3 Operational Definition	9
1.4 Research Question	10
1.5 Study Objectives	11
1.**5**.1 General Objective	11
1.**5**.2 Specific Objectives	11
**CHAPTER II**: LITERATURE REVIEW	12-14
**CHAPTER III**: RESEARCH METHODOLOGY	15-23
3.1 Study Design	15
3.2 Conceptual Framework	15
3.3 Target Population and Sampling Population	16
3.4 Sampling Unit and Elementary Unit	16
3.5 Study Period	16
3.6 Sample Size	16

3.7 Sampling Technique	17
3.8 Eligibility Criteria	17
3.9 Data Collection Mechanism	18
3.10 Survey Instrument	18
3.11 Data Management &Analysis Plan	19-20
3.12 Quality Control & Quality Assurance	20
3.13 Ethical Considerations	20
3.14 Expected Outcomes	21
3.15 Work Plan	21
References	22-26

ABBREVIATIONS

PSQ: Patient Satisfaction Questionnaire

FDMN: Forcibly Displaced Myanmar Nationals

MODMR: Ministry of Disaster Management and Relief

MOHFW: Ministry of Health and Family Welfare

IRB: Institutional Review Board

RRRC: Refugee Relief and Repatriation Commissioner

SPSS : Statistical Package for the Social Sciences

SD: Standard Deviation

OSAT: Overall Satisfaction

SE: Standard Error

CHAPTER I
INTRODUCTION

1.1 Introduction

The term “patient satisfaction” is conceptualized as critical touchstone for standard in healthcare sector in terms of consumer retention as well as status of service quality [1]. Review from the standpoint of patients is crucial in understanding the perceived standard of health system [2]. Highly satisfied patients are more empowered, efficient, dedicated to care and adhered to treatment resulting in desired improvement in health outcome along with reduced possibility of dropout from the course of medical management [3-5]. In consideration with subjectivity, factors including sex, age and literacy level of patients, quality of nursing, amount of time spent in the waiting space, communication with healthcare providers, provision of hospital facilities and result of treatment affect the scale of patient satisfaction to a great extent [6-8].

The arena of patient care has observed a transformation towards people-centric approach as the contentment of patients has made a point of significance equally with original goal of healing from morbidity [9]. The wording ‘Patient’ is contemplated for playing down the value of individual by tacitly generating the sense of social grading [10]. Market-oriented health economy recommends redefining the patient as a consumer which replaces traditional submissiveness from the view of patient to clinical institution with respectful intrapersonal synergy among patients and health care providers [11, 12]. In this regard, structured assessment on patient satisfaction is considered an instrumental tool to develop evidence-based recommendations for policymakers and relevant stakeholders to formulate and implement strategies directed toward the goal of satisfying the patient as a consumer of health services [13].

Bangladesh, which is one of the densely populated countries of the world with 164 million people, is providing shelter to 965,467 Rohingya population including 927,027 Forcibly Displaced Myanmar Nationals (FDMNs) and 38,440 refugees. Demographically, with 52% females and 48% males, the Rohingyas are constituted of 52% children, 44% adults and 4% older persons having an average family size of 5 people [14]. Led by several ministries (MODMR and MOHFW) of Bangladesh Government, 77 health sector partners are providing essential health services in Rohingya humanitarian emergency through a network

of 117 primary health care facilities [15]. In form of humanitarian assistance, the healthcare services are provided free of cost to Rohingya displaced beneficiaries with the support of different national and international partners.

Regarding satisfaction of host community patients in Bangladesh, several research were conducted and published focusing on maternal health, child health, family planning and diabetes mellitus [16]. Although, department of outdoor services is the maiden exposure to service availability of a health care center, very few studies were done to analyze the determining factors of client satisfaction in the regard. In addition, there is limited availability of published articles on satisfaction of patients from Rohingya displaced population hosted by Bangladesh. It is expected that, in Rohingya humanitarian emergency context, the proposed cross-sectional study will generate in-depth understanding of patients' perspective on outpatient healthcare in a primary health care facility and aid the relevant authority to improve satisfaction level of patients through coordinated initiatives.

1.2 Justification of the Study

Extensive literature review reveals scarcity of published research articles on satisfaction level among patients receiving healthcare services in Rohingya camps of Bangladesh. Moreover, information related to outpatient health services in Rohingya settings and associated satisfaction of beneficiaries were found with limited availability. Without substantial evidence on outpatient satisfaction care in health facilities placed in Rohingya camps, it is challenging for policymakers to identify service gaps and formulate recommendations for improving quality of healthcare centers. Considering this dearth of research activities, it is aimed to understand status of patient satisfaction and explore associated underlying factors in the context of outpatient department of a primary health care facility placed in Rohingya displaced camp. The study findings may act as a valuable resource in the future as these will guide policy and decision-makers to strengthen outpatient health service delivery prioritizing personalized needs of patients receiving healthcare in Rohingya camps.

1.3 Operational Definitions

Patient: A person with illness or injury requiring healthcare attention by a medical doctor, nurse, medical assistant, dental surgeon, counsellor, or any category of health worker.

Patient satisfaction: The indicator to evaluate the effectiveness and quality of service received by a patient in the healthcare system.

Primary health care facility: Health facility providing continuum of care from health promotion and disease prevention to treatment, rehabilitation and palliative care, and as close as feasible to people's everyday environment

Out-patient / outdoor department: The division of hospital treating patients who do not necessarily require admission in hospital to stay overnight at the time of consultation.

Rohingya: A Muslim ethnic minority group who have lived for centuries in predominantly Buddhist Myanmar - formerly known as Burma

Refugee: Refugees are people who have been forced to flee their homes and have crossed an international border to find safety in another country.

FDMN: The Rohingya population who fled from Myanmar in August 2017, are defined as Forcibly Displaced Myanmar Nationals (FDMNs) by Government of Bangladesh

1.4 Research Question (s)

a. What is the level of satisfaction in different dimensions among patients receiving outpatient care from primary healthcare facilities in Rohingya camps in Bangladesh?

b. Is there any difference in patient satisfaction across sociodemographic characteristics and health status of the beneficiaries regarding outpatient care in primary healthcare facilities in Rohingya camps in Bangladesh?

c. What are the key areas of dissatisfaction among patients with outpatient care from primary healthcare facilities in Rohingya camps in Bangladesh?

1.5 Study Objectives

1.5.1 General Objective

To assess patient satisfaction on outpatient services provided in a primary healthcare facility in Rohingya camps in Bangladesh.

1.5.2 Specific Objectives

a. To assess relationship of patient satisfaction with sociodemographic characteristics of the beneficiaries receiving outpatient care
b. To understand relation between types of health illness and facility visits with patient satisfaction on outpatient services
c. To explore key areas of dissatisfaction among patients received outpatient health care

CHAPTER II

LITERATURE REVIEW

Regarding the debate of engaging medical care consumers in assessing the quality of healthcare provided, Davies and Ware (1988), concluded that an authentic review of quality can be provided by consumers and relevant bias from individual traits are less significant enough for invalidating feedback of consumers [16]. Assessment of client satisfaction was stated as guiding evidence for authorities in identifying clients, setting performance indicators and developing management information systems according to Langseth et al., (1995) [17]. Exercise on patient satisfaction was considered a significant instrument by Donabedian (1988) for evaluating the quality, design and management of services provided in the health sector [1]. In a study conducted by Murphy-Cullen and Larsen (1984), the satisfaction of patients was found closely associated with improved treatment compliance, timeliness in health-seeking behaviour and enhanced retention of medical information [18].

De Silva et al., (2000) ranged the coverage of patient satisfaction with both medical and non-medical perspectives of care which can be measured by several established surveys including Patient Satisfaction Questionnaire (PSQ), CAHPS Questionnaire, Community Tracking Study, Hadad et al., Evaluation Ranking Scale Picker Commonwealth Survey of Patient Centred Care and QUOTE Survey [10]. Among these methods of assessing patient satisfaction, Wilkin et al., (1992) stated PSQ as arguably the most well-articulated and thoroughly tested tool [19].

In 2012, survey conducted in general outpatient departments in National Hospital Abuja of Nijeria found considerable relationship between a short waiting time and expectations fulfilled by patients during clinic visits [20]. In same year’s another study conducted on patient satisfaction in Iraq, researchers found comparatively higher levels of satisfaction in health beneficiaries from young, rural, married, unemployed, low education and good health backgrounds [21].

Multiple types of research were conducted in South Asia to explore factors influencing satisfaction levels on different provisions of health services [22]. A study carried out on allopathic health centers of India in 2009, 64.6% of patients expressed satisfaction regarding duration of the outdoor consultation department [23]. Similarly, another research in 2010 on maternal and child health services in rural areas of India revealed highly

satisfactory observations with more than 70% positive responses both for doctors and nursing staff [24]. In 2016, researchers found nearly the same level of overall satisfaction (73.1%) among urban healthcare beneficiaries of India with concerns expressed regarding amount of time spent with physicians [25]. A similar study also revealed high level of patient satisfaction in an Indian referral health facility; however, satisfaction level was found lowest in terms of accessibility and convenience [26]. Interestingly, for psychiatric patients, level of satisfaction varied greatly in accordance with different diagnostic categories as per the findings from a study of 2012 conducted in mental health outdoor department of a medical college in India. Patients diagnosed with depression came up with highest level of satisfaction, whereas schizophrenic patients expressed least satisfaction [27].

From a study carried among outdoor diabetic patients in Pakistan, it was found that satisfaction of patients was highly associated with their understandings on technical skills, interpersonal aspects and appropriateness of time provision [28]. Significant variation in levels of satisfaction was observed regarding category of health facilities of Pakistan in 2020; 86% patients in health facilities of public sector expressed confidence on perfection of received medical care, whereas 96% beneficiaries were confident in health centers of private sector [29]. In another survey on outdoor consultations in Pakistan in 2020, time spent with physicians and communication, were the two areas with lower patient satisfaction expressed [30]. In similar research conducted in Nepal in 2020, issue of availability and access to healthcare staff was raised with concern among study patients [31]. Another patient satisfaction study from Nepal in 2021 found that study patients expressed 39%, 45% and 92% satisfaction respectively on dimension regarding general satisfaction, accessibility and convenience, and interpersonal manner [32].

In the ’90s decade, several studies were carried out in government health facilities of Bangladesh to understand the level of client satisfaction with family planning services [33-40]. Notably, a linear association between the satisfaction level of clients and the quality of family planning services provided in these health centers was not observed in a consistent manner [41]. Different dimensions of service quality and its association with the satisfaction of patients were analyzed by Andaleeb (2001) for hospitals in Bangladesh [42]. In this study, discipline in the service environment and assurance of hospital employees were found to be more important dimensions for patient satisfaction than responsiveness and communication. Aldana et al., (2001) indicated the behaviour of government service

providers as a stronger predictor of patient satisfaction than the technical capacity of service providers in rural Bangladesh [41]. In a similar study, Andaleeb et al., (2007) suggested medical doctors' orientation as the most powerful factor to impact the satisfaction level of patients in hospital settings of Bangladesh [43].

The comparative study on service quality and predictors of hospital choice for public and private hospitals in Bangladesh, done by Andaleeb (2000) revealed a higher evaluation for private health facilities regarding responsiveness, communication, and discipline [44]. Regarding public hospitals of Bangladesh, patient expectations were explored by Rahman et al., (2002), which included upgradation of treatment facilities, improvement in medicine supply, strengthening of emergency, pathology and other departments, shift in staff attitude of health centers, increase in doctors' attention to patients, recruitment of skilled human resource and well maintenance of housekeeping [45]. For improving satisfaction level of patients in Bangladesh, minimization the waiting time for patients was underscored with more significance than the increase in shorter physician consultation time for them [41]. Poor adherence to treatment and missing follow-up care were identified as key effects resulting from the dissatisfaction of patients [46]. In another patient satisfaction study conducted among inpatient and outdoor patients of Bangladesh in 2002, researchers found higher satisfaction among youths, faced with shorter waiting time and patients with less education [47].

CHAPTER III

RESEARCH METHODOLOGY

3.1 Study Design

The cross-sectional method will be used to design this survey.

3.2 Conceptual Framework

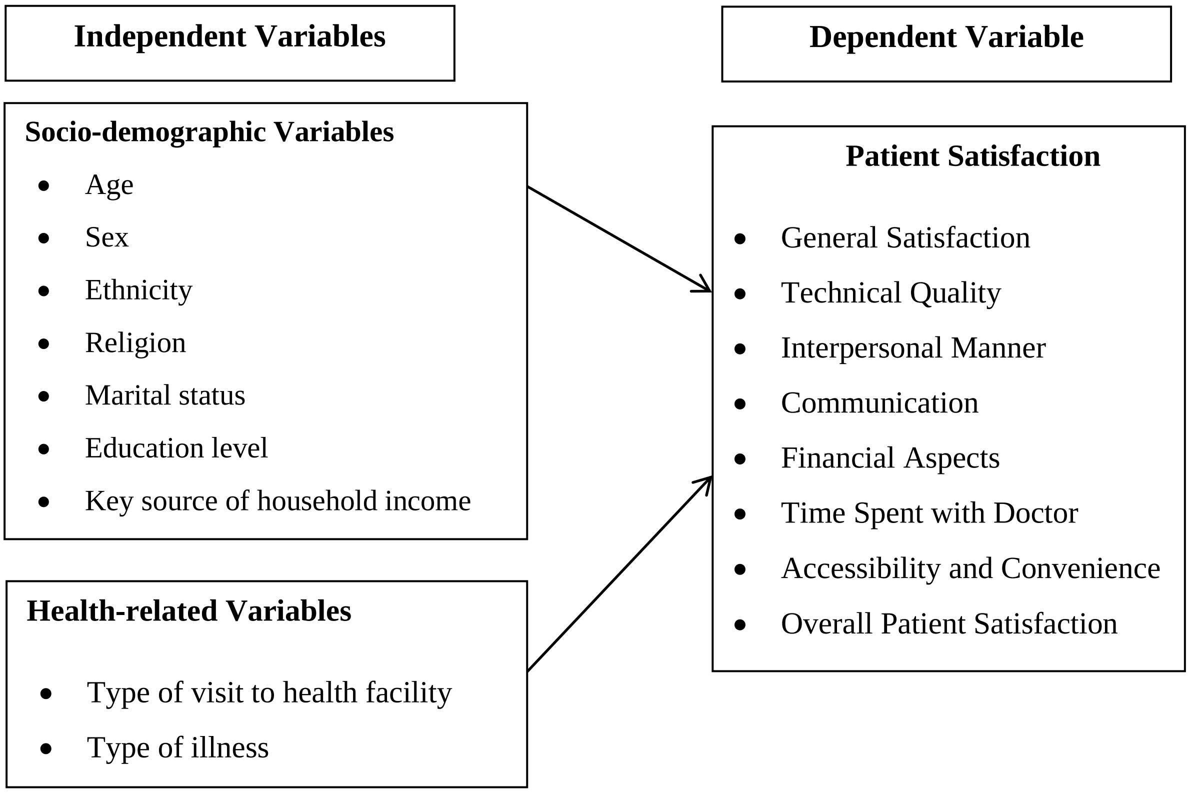

3.3 Target Population and Sampling Population

Irrespective of sex and age, the target population for this research will be all persons living in Rohingya camps and adjacent host community areas in Cox's Bazar, Bangladesh. Among the targeted people, the sampling population will be the beneficiaries visiting outpatient departments of primary health care facilities placed in Rohingya camps of Cox's Bazar.

3.4 Sampling Unit and Elementary Unit

As sampling frame, the list of functional health facilities in Rohingya camps will be collected from local government health authority. One health facility will be selected from this list as a sampling unit. The patient receiving outdoor health service from the selected health facility will be considered as an elementary unit in this survey.

3.5 Study Period

Study duration will be from 15 November 2023 to 15 March 2024.

3.6 Sample Size

Estimation of the sample size has been done using the formula, n= (z^2 pq)/d^2

Here,

n = Sample size

z = z-value at a given level of confidence level (for 97% confidence level, z = 2.17)

p = Expected prevalence of patient satisfaction (it is assumed as 50% / 0.50)

q = 1-p = 1-0.50 = 0.50

d = Margin of error is estimated as 4%

Adding expected 10% of incomplete responses, the required sample size (n) will be 810.

3.7 Sampling Technique

Two-stage random sampling technique will be used in this study. Firstly, five primary health care facilities in Rohingya camps will be randomly as study centers situated across five different Rohingya camps (2 West, 8 West, 10, 13 and 16). Secondly, random sampling method will be used to select the outdoor patients of selected health facility.

3.8 Eligibility Criteria

Inclusion Criteria:

1. All adult patients (aged ≥18 years) visiting health facility to receive outdoor consultations of selected health facility
2. Patients willing to participate and give consent
3. Understand the study questionnaire

Exclusion Criteria:

1. Patients with emergency health conditions
2. Patients aged below 18 years
3. Patients refusing to provide consent
4. Patients interviewed previously during the period of data collection
5. Healthcare staff of the health facility where data is being collected
6. Fail to understand the study questions and communicate orally

3.9 Data Collection Mechanism

Through face-to-face interviews, data will be collected using a predesigned paper-based structured questionnaire. The questionnaire will be translated to Bengali language for ease of communication. To predict operational challenges and identify limitations, piloting will be done taking 10% of the estimated sample size, who will not be included in the study.

3.10 Survey Instrument

The research questionnaire will be comprised of four segments. Starting with basic information (name and address) in the first segment, data on sociodemographic variables (age, sex, ethnicity, religion, marital status, education level and employment) of patients will be collected using the second section of the questionnaire. The next segment will gather data on health status of patients including types of facility visit and category of illness. In the final section, a globally recognized survey tool, the Patient Satisfaction Questionnaire (PSQ-18) will be used to assess satisfaction level of patients [48]. Utilizing a total of 18 question items, the PSQ-18 evaluates patient satisfaction in seven sub-scales which include general satisfaction, technical quality, interpersonal manner, communication, financial aspects, time spent with the doctor, and accessibility and convenience. Findings of total 18 questions will be analysed from PSQ-18, where each question will yield five options for study participants including strongly agree, agree, uncertain, disagree and strongly disagree. All items were scored from one to five so that high scores reflect satisfaction with health care. After item scoring, items within each scale will be averaged together to create the seven subscale scores.

3.11 Data Management & Analysis Plan

Following collection of all responses form paper-based questionnaires, the data will be entered and analyzed in IBM Statistical Package for the Social Sciences (SPSS) version 27 (IBM Corporation, Armonk, NY, USA). Information regarding sociodemographic and health variables will be presented in frequencies and percentages.

The question items of PSQ-18 will yield separate scores for each of seven domains; general satisfaction (items 3 and 17), technical quality (items 2, 4, 6 and 14), interpersonal manner (items 10 and 11), communication (items 1 and 13), financial aspects (items 5 and 7), time spent with doctor (items 12 and 15), accessibility and convenience (items 8, 9, 16 and 18), alongside overall satisfaction (all 18 items).

Scoring for all question items will be done for reflecting satisfaction with medical care by high scores as per the below table [48].

Question Item Numbers	Original Response Value	Scored Value
1, 2, 3, 5, 6, 8, 11, 15, 18	1 --------------->	5
	2 --------------->	4
	3 --------------->	3
	4 --------------->	2
	5 --------------->	1
4, 7, 9, 10, 12, 13, 14, 16, 17	1 --------------->	1
	2 --------------->	2
	3 --------------->	3
	4 --------------->	4
	5 --------------->	5

Tests for normality of data will be conducted for dependent variables using Kolmogorov-Smirnov test and Shapiro-Wilk test. To check internal consistency of survey questionnaire, reliability tests will be utilized to measure Cronbach's alpha. Descriptive statistics will be used for generating mean, standard deviation and percentage for all dimensions of patient satisfaction including items of PSQ-18 questionnaire. Finally, mean difference in patient satisfaction across independent and dependent variables will be explored through nonparametric Kruskal–Wallis test with significance level 0.05 and comparison of means.

3.12 Quality Control & Quality Assurance

For checking quality and standards during study, the researcher will review the collected data and compile final report. With the required guidance from the Department of Public Health, North South University, standard quality will also be maintained during data collection and report presentation. Overall, the study investigator will review the quality of data during collection, entry, compilation, and analysis.

3.13 Ethical Considerations

All required ethical and administrative approvals will be obtained respectively from the IRB of North South University and RRRC (Refugee Relief and Repatriation Commissioner) office, Cox's Bazar. Before undertaking interviews, written informed consent will be obtained from each willing respondent ensuring respect for right to informed consent, right to deny and right to accurate representation. Although the consent form will be in English language, it will be read out and explained in detail to study participants in local dialect. Voluntary nature of participation will be informed to every respondent and option will be given to withdraw from investigation at any time, without justification, and without any consequence. All collected information will be handled with utmost confidentiality with use limited to only research purpose.

3.14 Expected Outcomes

For humanitarian emergency settings like Rohingya response, evidence will be generated about status of patient satisfaction across multiple domains and influence of sociodemographic as well as health related factors on different aspects of patient satisfaction in Rohingya hum. Relevant findings regarding the domains resulted in low patient satisfaction, will be shared with policy and decision makers for developing domain specific targeted interventions on priority basis. Recommendations for future research designs on this subject will also be discussed in this study.

3.15 Work Plan

Starting from 15 November 2023, different components of the proposed study are planned to be completed by 31 March 2024. The study components consist of literature review, designing of study, ethical and administrative approval, data collection, entry, analysis, report writing, defense and submission of thesis.

REFERENCES

1. Donabedian A. The quality of care: how can it be assessed?. Jama. 1988 Sep 23;260(12):1743-8.

2. Fufa BD, Negao EB. Satisfaction of outpatient service consumers and associated factors towards the health service given at Jimma Medical Center, South West Ethiopia. Patient related outcome measures. 2019;10:347.

3. Pascoe GC. Patient satisfaction in primary health care: a literature review and analysis. Evaluation and program planning. 1983 Jan 1;6(3-4):185-210.

4. DuPree E, Anderson R, Nash IS. Improving quality in healthcare: start with the patient. Mount Sinai Journal of Medicine: A Journal of Translational and Personalized Medicine. 2011 Nov;78(6):813-9.

5. Saisho Y. Use of diabetes treatment satisfaction questionnaire in diabetes care: importance of patient-reported outcomes. International journal of environmental research and public health. 2018 May;15(5):947.

6. Schoenfelder T, Klewer J, Kugler J. Determinants of patient satisfaction: a study among 39 hospitals in an in-patient setting in Germany. International journal for quality in health care. 2011 Oct 1;23(5):503-9.

7. Pai Y, Ravi G, Chary S. Factors affecting In-patient Satisfaction in Hospital-A Case Study. InDubai: Proceedings of the 2011 International Conference on Technology and Business Management (ICTBM-11) 2011.

8. Al-Abri R, Al-Balushi A. Patient satisfaction survey as a tool towards quality improvement. Oman medical journal. 2014 Jan;29(1):3.

9. Jan MU, Hassan Z, Khan MS, Ullah R, Siraj A. Comparison of Patient Satisfaction Level and its Various Determinants in Public and Private Hospitals in Peshawar. Dr. Sulaiman Al Habib Medical Journal. 2020;2(4):167-73.

10. De Silva A, Valentine N. A framework for measuring responsiveness. World Health Organization GPE Discussion Paper Series. 2000.

11. Owens DJ, Batchelor C. Patient satisfaction and the elderly. Social science & medicine. 1996 Jun 1;42(11):1483-91.

12. Sitzia J, Wood N. Patient satisfaction: a review of issues and concepts. Social science & medicine. 1997 Dec 1;45(12):1829-43.

13. Farooq A, Khaliq MA, Toor MA, Amjad A, Khalid W, Butt F. Assessment of Patient Satisfaction in a Military and Public Hospital: A Comparative Study. Cureus. 2020 Aug 31;12(8).

14. Rohingya Refugee Response/Bangladesh: Joint Government of Bangladesh - UNHCR Population Factsheet. Retrieved from: https://data.unhcr.org/en/documents/details/103840

15. Health Sector Cox's Bazar Monthly Bulletin - September 2023. Retrieved from: https://rohingyaresponse.org/sectors/coxs-bazar/health/

16. Adhikary G, Shawon MS, Ali MW, Shamsuzzaman M, Ahmed S, Shackelford KA, Woldeab A, Alam N, Lim SS, Levine A, Gakidou E. Factors influencing patients' satisfaction at different levels of health facilities in Bangladesh: Results from patient exit interviews. PloS one. 2018 May 16;13(5):e0196643.

16. Davies AR, Ware Jr JE. Involving consumers in quality of care assessment. Health affairs. 1988;7(1):33-48.

17. Langseth P, Langan P, Talierco R. Service delivery survey (SDS): a management tool. The Economic Development Institute of the World Bank. 1995.

18. Murphy-Cullen CL, Larsen LC. Interaction between the socio-demographic variables of physicians and their patients: its impact upon patient satisfaction. Social Science & Medicine. 1984 Jan 1;19(2):163-6.

19. Wilkin D, Hallam L, Doggett M. Measures of patient satisfaction. Measures of need and outcome for primary health care. 1992:230-8.

20. Ogunfowokan O, Mora M. Time, expectation and satisfaction. Patients' experience at National Hospital Abuja, Nigeria. Afr J Prim Health Care Fam Med 2012;4:6. Available from: http://www.dx.doi. org/10.4102/ phcfm.v4i1.398. [Last accessed on 2014 Nov 17]

21. Jadoo, S. a. A., Yaseen, S. M., Al-Samarrai, M. a. M., & Mahmood, A. S. (2020). Patient satisfaction in outpatient medical care: the case of Iraq. Journal of Ideas in Health, 3(2), 176–182. https://doi.org/10.47108/jidhealth.vol3.iss2.44

22. Sivalenka S. Patient Satisfaction Surveys in Public Hospitals in India. Available from: http://www.rand.org. [Last accessed on 2014 Nov 24]

23. Kumari R, Idris M, Bhushan V, Khanna A, Agarwal M, Singh S. Study on patient satisfaction in the government allopathic health facilities of Lucknow district, India. Indian J Community Med 2009;34:35-42

24. Das P, Basu M, Tikadar T, Biswas G, Mridha P, Pal R. Client satisfaction on maternal and child health services in rural Bengal. Indian J Community Med 2010;35:478-81.

25. Chakraborty SN, Bhattacherjee S, Rahaman MA. A cross-sectional study on patient satisfaction in an Urban Health Care Centre of Siliguri Municipal Corporation, Darjeeling, West Bengal. Med J DY Patil Univ 2016;9:325-30.

26. Gaur BP, Jahnavi G, Thatkar PV. Patient satisfaction about services obtained from a teaching hospital, Port Blair: A cross-sectional study. J Family Med Prim Care 2020;9:93-8.

27. Holikatti PC, Kar N, Mishra A, Shukla R, Swain SP, Kar S. A study on patient satisfaction with psychiatric services. Indian J Psychiatry 2012;54:327-32.

28. Jalil, A., Zakar, R., & Zakar, M. Z. (2018). Satisfaction of diabetes patients in public outpatient department: prevalance and determinants. Rawal Medical Journal, 43(1), 8–13. https://www.ejmanager.com/fulltextpdf.php?mno=259165

29. Jan, M. U., Hassan, Z., Khan, M. S., Ullah, R., & Siraj, A. (2020). Comparison of Patient Satisfaction Level and its Various Determinants in Public and Private Hospitals in Peshawar. Dr. Sulaiman Al Habib Medical Journal, 2(4), 167. https://doi.org/10.2991/dsahmj.k.200903.001

30. Sandhu, A. I., & Liaqat, N. (2020). Patient satisfaction Questionnaire: A tool towards improvement of Healthcare services. ResearchGate. https://www.researchgate.net/publication/338675080

31. Poudel, L., Baskota, S., Mali, P., Pradhananga, P., Malla, N., Rajbhandari, B., & Nepal, S. (2020). Patient satisfaction in out-patient services at a tertiary care center: a descriptive cross-sectional study. Journal of Nepal Medical Association, 58(225). https://doi.org/10.31729/jnma.4917

32. Adhikari, M., Paudel, N. R., Mishra, S. R., Shrestha, A., & Upadhyaya, D. P. (2021). Patient satisfaction and its socio-demographic correlates in a tertiary public hospital in Nepal: a cross-sectional study. BMC Health Services Research, 21(1). https://doi.org/10.1186/s12913-021-06155-3

33. Whittaker M. Rural women's perspectives on quality of family planning services. Dhaka, Bangladesh, ICDDR. B, 1993 (Working Paper No. 85 MCH-FP Extension Project); 1993.

34. Khanun P. Service delivery at the union health and family welfare centers: the client's perspective. Dhaka, Bangladesh, ICDDR. B, 1994 (Working Paper No. 110, MCH-FP Extension Project); 1994.

35. Mabub F, Huq Md N, Rashid MA. An assessment of counselling for clinical FP methods in GOB clinics. Dhaka, National Institute of Population Research and Training. 1991.

36. Hashemi SM. Evaluation of Knowledge and Skills of Field Level Workers of Health and Family Planning Programmes. Population Development and Evaluation Unit, Implementation Monitoring and Evaluation Division, Ministry of Planning; 1995.

37. Al-Sabir A. Evaluation of FWVs skills as MCH-FP services providers in Bangladesh. Dhaka, National Institute of Population Research and Training. 1995.

38. Hossain MB, Mita R, Haaga JG. Quality of care and contraceptive adoption in rural Bangladesh: MCH-FP extension project areas. Dhaka, Bangladesh, ICDDR. B, 1991 (Working Paper No. 61, MCH-FP Extension Project); 1991.

39. Hasan M. Accessibility and services in satellite clinics: findings from exit interview. ICDDR, E MCH-FP Extension Project (Rural) Working Paper. 1994 Jan(98).

40. Kamal GM. The quality of NORPLANT services in Bangladesh. Associates for Community and Population Research; 1991.

41. Aldana JM, Piechulek H, Al-Sabir A. Client satisfaction and quality of health care in rural Bangladesh. Bulletin of the World Health Organization. 2001;79:512-7.

42. Andaleeb SS. Service quality perceptions and patient satisfaction: a study of hospitals in a developing country. Social science & medicine. 2001 May 1;52(9):1359-70.

43. Andaleeb SS, Siddiqui N, Khandakar S. Patient satisfaction with health services in Bangladesh. Health policy and planning. 2007 Jul 1;22(4):263-73.

44. Andaleeb SS. Public and private hospitals in Bangladesh: service quality and predictors of hospital choice. Health policy and planning. 2000 Mar 1;15(1):95-102.

45. Rahman MM, Shahidullah M, Shahiduzzaman M, Rashid HA. Quality of health care from patient perspectives. Bangladesh Medical Research Council Bulletin. 2002 Dec 1;28(3):87-96.

46. Andaleeb SS, Siddiqui N, Khandakar S. Patient satisfaction with health services in Bangladesh. Health policy and planning. 2007 Jul 1;22(4):263-73.

47. MM Rahman, M Shahidullah, M Shahiduzzaman, HA Rashid. Quality of Health Care from Patient Perspectives. Bangladesh Med. Res. Counc. Bull. 2002; 28 (3): 87-96

48. Marshall GN, Hays RD. The Patient Satisfaction Questionnaire Short-form (PSQ-18). Santa Monica, California: RAND; 1994.
