# Supplementary material for: Patient satisfaction in outdoor department of primary health care facilities in Rohingya refugee camps in Bangladesh: A cross-sectional study
Source: PLoS One. 2026 Jan 13;21(1):e0336811. doi: 10.1371/journal.pone.0336811 (PMC12798992; doi:10.1371/journal.pone.0336811)
Supplement: S3 File — (PDF) [file pone.0336811.s003.pdf]

Patient Satisfaction Questionnaire

Code Number: Date of Interview:

Starting Time of Interview: End Time of Interview:

Place of Interview:

✂--

Section 1: Basic Information

Instruction: Write information in designated spaces in English language
(নির্দেশাবলী: ইংরেজি ভাষায় নির্দিষ্ট জায়গায় তথ্য লিপিবদ্ধ করুন)

B1 Name of Patient (রোগীর নাম):
B2 Progress ID (প্রগ্রেস আইডি) / National ID (জাতীয় পরিচয়পত্র নং):
B3 Block (ব্লক) / Village (গ্রাম)::
B4 Camp (ক্যাম্প) / Union (ইউনিয়ন):
B5 Upazilla (উপজেলা):
B6 Name of Househead (বাড়ীর প্রধান ব্যক্তির নাম):
B7 Name of Majhi (মাঝির নাম):

✂--

Section 2: Sociodemographic Factors

Instruction: Provide tick mark in right option (নির্দেশাবলী: সঠিক জায়গায় টিক চিহ্ন দিন)

S1 Age (Year) বয়স (বছর):

S2 Sex (লিঙ্গ): □ Male (পুরুষ) □ Female (মহিলা) □ Other (অন্যান্য)

S3 Ethnicity (জাতি): □ Rohingya (রোহিঙ্গা) □ Bangladeshi (বাংলাদেশী)

S4 Religion (ধর্ম): □ Islam (ইসলাম) □ Hindu (হিন্দু)

□ Christian (খৃস্টান) □ Others (অন্যান্য)

S5 Marital status: (বৈবাহিক অবস্থা) □ Unmarried (অবিবাহিত) □ Married (বিবাহিত)

□ Divorced (বিবাহবিচ্ছেদ) □ Widowed (বিপত্নীক/বিধবা)

Patient Satisfaction Questionnaire

Code Number:

Instruction: Provide tick mark in right option (নির্দেশাবলী: সঠিক জায়গায় টিক চিহ্ন দিন)

**S6**	**Educational level:** **(শিক্ষাগত যোগ্যতা)**	□ No Literacy (Can not read or write a letter) অক্ষরজ্ঞানহীন (চিঠি পড়তে বা লিখতে পারে না)<br><br>□ Basic Literacy (Can read a letter) প্রাথমিক সাক্ষরতা (চিঠি পড়তে পারে)<br><br>□ Functional Literacy (Can read and write a letter) কার্যকর সাক্ষরতা (চিঠি পড়তে ও লিখতে পারে)	
**S7**	**Key source of household income:** **(পরিবারের উপার্জনের মূল উৎস)**	□ Daily Labour (দিনমজুর)	□ Small Businees (ক্ষুদ্র ব্যবসা)
		□ NGO Volunteer (এনজিও স্বেচ্ছাসেবী)	□ Masonry (রাজমিস্ত্রী)
		□ Tailor (সেলাই দর্জি)	□ Teacher (শিক্ষক)
		□ Others (অন্যান্য)	□ Depends only on humanitarian aids (কেবলমাত্র মানবিক সহায়তার উপর নির্ভরশীল)

Section 3: Health Related Factors

Instruction: Provide tick mark in right option (নির্দেশাবলী: সঠিক জায়গায় টিক চিহ্ন দিন)

**H1**	**Type of Visit in Health Facility:** (স্বাস্থ্যকেন্দ্রে আগমনের ধরন)	□ New Visit (প্রথমবার আগমন)	□ Follow-up Visit (চিকিৎসার অগ্রগতি জানার জন্য আগমন)
**H2**	**Type of Illness** (অসুস্থতার ধরন)	□ Acute (আকস্মিক)	□ Chronic (দীর্ঘমেয়াদী)

Patient Satisfaction Questionnaire

Code Number:

Section 4: Patient Satisfaction Factors

Instruction: Circile one number in each line
(নির্দেশাবলী: সঠিক নাম্বার গোল দাগ দিয়ে চিহ্নিত করুন)

We would like to know about your feelings, good and bad, about the medical care you received. How strongly do your agree or diagree with each of the following statements? (আপনি যে চিকিৎসা সেবা গ্রহণ করলেন, তা সম্পর্কে আপনার অনুভূতি (ভালো ও খারাপ) জানতে চাই। নিম্নলিখিত মন্তব্যগুলোর সাথে আপনি কি পরিমাণ দৃঢ়তার সাথে একমত বা ভিন্নমত পোষণ করেন?)

Sl. No.	*Statements (মন্তব্যসমূহ)*	*Strongly Agree (দৃঢ়ভাবে একমত)*	*Agree (একমত)*	*Uncertain (দ্বিধাগ্রস্ত)*	*Disagree (ভিন্নমত)*	*Strongly Disagree (দৃঢ়ভাবে ভিন্নমত)*
1	Doctors are good about explaining the reason for medical tests (চিকিৎসকগণ ভালোভাবে রোগ নীরিক্ষার কারণ ব্যাখ্যা করেন)	**1**	**2**	**3**	**4**	**5**
2	I think my doctor's office has everything needed to provide complete medical care (আমি মনে করি আমার চিকিৎসকের কর্মস্থলে সম্পূর্ণ চিকিৎসা সেবা প্রদানের জন্য প্রয়োজনীয় সব ব্যবস্থা আছে)	**1**	**2**	**3**	**4**	**5**
3	The medical care I have been receiving is just about perfect (আমি যে চিকিৎসা সেবা পাচ্ছি, তা প্রায় নিখুঁত)	**1**	**2**	**3**	**4**	**5**
4	Sometimes doctors make me wonder if their diagnosis is correct (মাঝে মাঝে চিকিৎসকরা আমাকে এই ভেবে অবাক করে যে আদৌ তাদের রোগ নির্ণয় ঠিক আছে কি না)	**1**	**2**	**3**	**4**	**5**

Patient Satisfaction Questionnaire

Code Number:

Instruction: Circile one number in each line
(নির্দেশাবলী: সঠিক নাম্বার গোল দাগ দিয়ে চিহ্নিত করুন)

How strongly do your agree or diagree with each of the following statements?
(নিম্নলিখিত মন্তব্যগুলোর সাথে আপনি কি পরিমাণ দৃঢ়তার সাথে একমত বা ভিন্নমত পোষণ করেন?)

Sl. No.	*Statements (মন্তব্যসমূহ)*	*Strongly Agree (দৃঢ়ভাবে একমত)*	*Agree (একমত)*	*Uncertain (দ্বিধাগ্রস্ত)*	*Disagree (ভিন্নমত)*	*Strongly Disagree (দৃঢ়ভাবে ভিন্নমত)*
5	I feel confident that I can get the medical care I need without being set back financially (আমি এই ভেবে আত্মবিশ্বাসী যে, অর্থনৈতিকভাবে ক্ষতিগ্রস্ত হওয়া ছাড়াই আমি প্রয়োজনীয় চিকিৎসা সেবা পাবো)	**1**	**2**	**3**	**4**	**5**
6	When I go for medical care, they are careful to check everything when treating and examining me (যখন আমি চিকিৎসা সেবা গ্রহণ করতে যাই, তারা আমাকে চিকিৎসা এবং পরীক্ষা করার সময় সতর্কতার সাথে সবকিছু দেখে)	**1**	**2**	**3**	**4**	**5**
7	I have to pay for more of my medical care than I can afford (চিকিৎসা সেবা গ্রহণ করার জন্যে আমাকে সক্ষমতার চেয়ে বেশি অর্থ ব্যয় করা লাগে)	**1**	**2**	**3**	**4**	**5**
8	I have easy access to the medical specialists I need (আমি প্রয়োজন মোতাবেক বিশেষজ্ঞ চিকিৎসকদের কাছে সহজেই যেতে পারি)	**1**	**2**	**3**	**4**	**5**

Patient Satisfaction Questionnaire

Code Number:

Instruction: Circile one number in each line
(নির্দেশাবলী: সঠিক নাম্বার গোল দাগ দিয়ে চিহ্নিত করুন)

How strongly do your agree or diagree with each of the following statements?
(নিম্নলিখিত মন্তব্যগুলোর সাথে আপনি কি পরিমাণ দৃঢ়তার সাথে একমত বা ভিন্নমত পোষণ করেন?)

Sl. No.	*Statements (মন্তব্যসমূহ)*	*Strongly Agree (দৃঢ়ভাবে একমত)*	*Agree (একমত)*	*Uncertain (দ্বিধাগ্রস্ত)*	*Disagree (ভিন্নমত)*	*Strongly Disagree (দৃঢ়ভাবে ভিন্নমত)*
9	Where I get medical care, people have to wait too long for emergency treatment (যখন আমি চিকিৎসা সেবা গ্রহণ করি, জরুরী চিকিৎসা নিতে মানুষকে অনেক বেশি অপেক্ষা করতে হয়)	**1**	**2**	**3**	**4**	**5**
10	Doctors act too businesslike and impersonal toward me (চিকিৎসকরা আমার প্রতি খুবই ব্যবসায়িক এবং রুঢ় আচরণ করে)	**1**	**2**	**3**	**4**	**5**
11	My doctors treat me in a very friendly and courteous manner (আমার চিকিৎসকরা আমার সাথে অনেক বন্ধুত্বপূর্ণ এবং ভদ্র আচরণ করে)	**1**	**2**	**3**	**4**	**5**
12	Those who provide my medical care sometimes hurry too much when they treat me (যারা আমাকে চিকিৎসা সেবা প্রদান করে, তারা মাঝে মাঝে আমাকে সেবা দেওয়ার সময় অনেক তাড়াহুড়া করে)	**1**	**2**	**3**	**4**	**5**
13	Doctors sometimes ignore what I tell them (চিকিৎসকদের আমি যা বলি, তা তারা মাঝে মাঝে উপেক্ষা করে)	**1**	**2**	**3**	**4**	**5**

Patient Satisfaction Questionnaire

Code Number:

Instruction: Circile one number in each line
(নির্দেশাবলী: সঠিক নাম্বার গোল দাগ দিয়ে চিহ্নিত করুন)

How strongly do your agree or diagree with each of the following statements?
(নিম্নলিখিত মন্তব্যগুলোর সাথে আপনি কি পরিমাণ দৃঢ়তার সাথে একমত বা ভিন্নমত পোষণ করেন?)

Sl. No.	*Statements (মন্তব্যসমূহ)*	*Strongly Agree (দৃঢ়ভাবে একমত)*	*Agree (একমত)*	*Uncertain (দ্বিধাগ্রস্ত)*	*Disagree (ভিন্নমত)*	*Strongly Disagree (দৃঢ়ভাবে ভিন্নমত)*
14	I have some doubts about the ability of the doctors who treat me (যে চিকিৎসকরা আমাকে সেবা প্রদান করে, তাদের সক্ষমতা নিয়ে আমার কিছুটা সন্দেহ আছে)	**1**	**2**	**3**	**4**	**5**
15	Doctors usually spend plenty of time with me (চিকিৎসকরা সাধারণত আমার সাথে পর্যাপ্ত সময় ব্যয় করে)	**1**	**2**	**3**	**4**	**5**
16	I find it hard to get an appointment for medical care right away (চিকিৎসা সেবার জন্য তাৎক্ষনিক সাক্ষাৎকারের সুযোগ পেতে আমার কষ্ট হয়)	**1**	**2**	**3**	**4**	**5**
17	I am dissatisfied with some things about the medical care I receive (আমি যে চিকিৎসা সেবা গ্রহণ করি, তার কিছু বিষয় নিয়ে আমি অসন্তুষ্ট)	**1**	**2**	**3**	**4**	**5**
18	I am able to get medical care whenever I need it (আমার যখন চিকিৎসা সেবা দরকার তখনই পাই)	**1**	**2**	**3**	**4**	**5**

Name of Interviewer ……………………………………………………………………………….

Signature of Interviewer ……………………………………………………………………….

Date (mm/dd/yy):
