# Supplementary material for: Patient satisfaction in outdoor department of primary health care facilities in Rohingya refugee camps in Bangladesh: A cross-sectional study
Source: PLoS One. 2026 Jan 13;21(1):e0336811. doi: 10.1371/journal.pone.0336811 (PMC12798992; doi:10.1371/journal.pone.0336811)
Supplement: Attachment — Submitted filename: Review Comment.docx [file pone.0336811.s005.docx]

**Review Report for Manuscript PONE-D-24-55548**

**Title:** Patient Satisfaction in Outdoor Department of Primary Health Care Facilities in Rohingya Refugee Camps in Bangladesh: A Cross-Sectional Study

The manuscript reports on a study evaluating patient satisfaction in the primary healthcare facilities serving the Rohingya refugee camps in Bangladesh. The study employed a cross-sectional design, sampling 810 patients across five health centers with 723 completed responses analyzed. It leveraged the Patient Satisfaction Questionnaire Short Form (PSQ-18) to measure satisfaction across multiple domains. The study addresses an important and underexplored area of healthcare delivery in a crisis setting, providing current data that can inform ongoing health service provision in refugee camps. The use of a structured and validated tool (PSQ-18) to assess patient satisfaction and the application of robust statistical methods are major strengths, contributing to the reliability of the findings. The relatively large sample size and high response rate enhance the study's generalizability to the Rohingya refugee population in Bangladesh.

The abstract has been written nicely and right to the point. The introduction part contains a review of the literature on the issue. The results section is detailed and provides a clear insight into patient satisfaction in the outdoor department of primary health care facilities in Rohingya refugee camps. It successfully highlights various factors influencing patient satisfaction, such as socio-demographic factors, type of visit, and illness. The tables are informative and support the text effectively.

However, following my review, I would like to suggest that the paper needs some revisions, and I think some points need to be addressed before further publication processing. The review comments are given below:

1. In the abstract, specific statistical analysis, such as the “Kruskal–Wallis test” can be mentioned instead of the general term “nonparametric test.”
2. In the introduction section, the authors tried to review some related literature that lacks some basic background information. The authors could consider discussing the major factors contributing to patients’ satisfaction/dissatisfaction with medical consultations and services. Additionally, the current status of health status in Rohingya refugee camps and primary health care services and facilities should be addressed in the introduction section which will indicate the significance of the current study.
3. On page 3 (lines: 60-61) the authors mentioned “A prior study in Nigeria found considerable relationship between a short waiting time and expectations fulfilled by patients during clinic visits [5]”, but the present study didn’t examine the influence of waiting time on patients’ satisfaction instead it is used as one of the scales of the dependent variables. I suggest omitting this sentence and citing a recent study that focuses on various factors influencing patients' satisfaction in Bangladesh (Zakaria, M., Mazumder, S., Faisal, H. M., Zannat, R., Haque, M. R., Afrin, T., ... & Xu, J. (2024). Physician communication behaviors on patient satisfaction in primary care medical settings in Bangladesh. *Journal of Primary Care & Community Health*, *15*, [https://doi.org/10.1177/21501319241277](https://doi.org/10.1177/21501319241277396).)
4. The major part of the introduction section is written as: A prior study found… Similar research in Iraq revealed… A study on maternal and child health services in rural areas of India revealed… Researchers found… it was found that…which indicates poor English writing. The authors can revise the introduction part to improve the language quality.
5. In the method section, it is necessary to explain more how the authors utilized simple random sampling in selecting study participants. Based on the description provided, it seems more likely that the study employed a form of systematic sampling rather than pure simple random sampling during the second stage of selecting study participants.
6. This section should mention the validity of the scale, such as the translation process of the Patient Satisfaction Questionnaire (PSQ-18) from English to Bangla.
7. On page 6 (lines: 136-138) the author mentioned: “For all question items of PSQ-18, responses were structured across five types; ’Strongly Agree’, ‘Agree’, ‘Uncertain’, ‘Disagree’ and ‘Strongly Disagree’ with values ranging from 1 to 5 (See Table B in S1 File).” According to Table B in S1 File, it should state that for negative statements the scale value was reversed.
8. Existing literature suggests that the Rohingya people are often reluctant to talk with unknown people. How did the researchers handle the issue during data collection? What is the role the Majhi played in this regard?
9. Please rephrase the variable “type of illness” to “perception of the type of illness” since the study did not use medical reports to verify whether conditions were acute or chronic.
10. As the study participants are living in a humanitarian condition and refugee settings, it is expected that there is no significant variability in some of their characteristics. It is also difficult to change their socio-economic status in this current context. Accordingly, the authors could include some interventional variables such as health awareness, NGOs/INGOs intervention, or access to health information. I think it could be mentioned as one of the study’s limitations.
11. The study should perform a multivariate analysis like regression to identify significant predictors since bivariate analysis alone may not sufficiently report influential factors.
12. In the abstract and results section, correct the notation from p = <.001 to p < .001.
13. The surprisingly high patient satisfaction levels reported in this study, despite the ongoing struggles of the Rohingya with healthcare, might be explained by their extreme deprivation in their home country concerning basic health needs.
14. The discussion section should include a paragraph about the implications of the study findings.

Nonetheless, I put some suggestions, still, it is a good piece of erudite work and provides some insights into the patients’ satisfaction in the outdoor department of primary health care facilities in Rohingya refugee camps in Bangladesh that collected data from a large sample. Accordingly, I recommend this article be published after revisions.
