# Supplementary material for: Patient satisfaction in outdoor department of primary health care facilities in Rohingya refugee camps in Bangladesh: A cross-sectional study
Source: PLoS One. 2026 Jan 13;21(1):e0336811. doi: 10.1371/journal.pone.0336811 (PMC12798992; doi:10.1371/journal.pone.0336811)
Supplement: Attachment — Submitted filename: Response to reviewers.docx [file pone.0336811.s007.docx]

**Response to the Reviewer**

**Manuscript title: ‘Patient Satisfaction in Outdoor Department of Primary Health Care Facilities in Rohingya Refugee Camps in Bangladesh: A Cross-Sectional Study’**

**Manuscript reference number: PONE-D-24-55548**

We would like to thank the Academic Editor and respected reviewers for their constructive comments and guidance to improve the paper. Following up on the reviewers’ suggestions and recommendations, we have revised the manuscript, and each modification has been highlighted in red. We have modified the sections as the reviewers suggested. Our detailed response as per the clean copy of revised manuscript is found below.

**Reviewer Comment:**

**Author Response:**

**Reviewer: 1**

1. In the abstract, specific statistical analysis, such as the “Kruskal–Wallis test” can be mentioned instead of the general term “nonparametric test.”

**Response:** Thank you for this helpful suggestion which will enhance statistical precision. Accordingly, we have revised the abstract to explicitly mention the **Kruskal–Wallis test** instead of the general term “nonparametric test” in **Page 2, Line 41**

2. In the introduction section, the authors tried to review some related literature that lacks some basic background information. The authors could **consider discussing the major factors contributing to patients’ satisfaction/dissatisfaction with medical consultations and services.** Additionally, **the current status of health status in Rohingya refugee camps and primary health care services and facilities** should be addressed in the introduction section which will indicate the significance of the current study.

**Response:** Thank you for this constructive suggestion. In response, we have elaborated the introduction section in **Page 3, Lines 59-62,** highlighting the role of interpersonal care capacity and patient-centric behavioral skills of physicians, which have been shown to influence satisfaction more than social, economic and engagement factors. Relevant references have been added accordingly. In addition, as suggested, we reflected the current health status in Rohingya refugee camp in **Page 4, lines 92-95** based on recent Public Health Needs Assessment by Health Sector mentioning some key challenges - all of which can influence patient experience.

3. On page 3 (lines: 60-61) the authors mentioned “A prior study in Nigeria found considerable relationship between a short waiting time and expectations fulfilled by patients during clinic visits [5]”, but the present study didn’t examine the influence of waiting time on patients’ satisfaction instead it is used as one of the scales of the dependent variables**. I suggest omitting this sentence and citing a recent study that focuses on various factors influencing patients' satisfaction in Bangladesh** (Zakaria, M., Mazumder, S., Faisal, H. M., Zannat, R., Haque, M. R., Afrin, T., ... & Xu, J. (2024). Physician communication behaviors on patient satisfaction in primary care medical settings in Bangladesh. *Journal of Primary Care & Community Health*, *15*, [https://doi.org/10.1177/21501319241277](https://doi.org/10.1177/21501319241277396).)

**Response:** Thank you for this helpful suggestion. As guided, we have removed the sentence referencing the Nigerian study. Instead, in **Page 4, Lines 79-81**, we have cited the recommended study by Zakaria et al. (2024) into the section of the introduction where we discuss Bangladeshi literature related to patient satisfaction. This addition strengthens the Bangladesh-specific context of the literature review and supports the rationale for our study in primary health care.

4. The major part of the introduction section is written as: A prior study found… Similar research in Iraq revealed… A study on maternal and child health services in rural areas of India revealed… Researchers found… it was found that…which indicates poor English writing. The authors can revise the introduction part to improve the language quality.

**Response:** Thank you so much for pointing this out. We acknowledge that the earlier wording in the introduction section became repetitive and informal, damaging language quality. In response, we have thoroughly revised the relevant portion of the introduction to remove redundant phrases such as “a prior study found” and “it was found that” have been replaced with words focusing on the actual findings. The changes are reflected in **Page 3-4, Lines 66-79**.

5. In the method section, it is necessary to explain more how the authors utilized simple random sampling in selecting study participants. Based on the description provided, it seems more likely that the study employed a form of systematic sampling rather than pure simple random sampling during the second stage of selecting study participants.

Response: Thank you for the insightful comment. We acknowledge the information gap regarding our method of selecting patients as study participants. In our study, we employed systematic random sampling technique in this regard. A single-digit number (3) was randomly selected to determine the starting point, after which every third patient was enrolled. This explanation has been incorporated into the **Methodology section** **(Page 5, Lines 107-111)** as well in the **Abstract (Page 2, Line 36)**.

6. This section should mention the validity of the scale, such as the translation process of the Patient Satisfaction Questionnaire (PSQ-18) from English to Bangla.

**Response:** Thank you for the valuable suggestion. We have clarified the translation process in the revised manuscript in **Page 6, Lines 138-141**. Linguistic experts within our research team, with prior experience working with the PSQ-18 questionnaire, were involved in the Bengali translation of the tool. Finally, this was finalized through team consensus for ensuring contextual consistency.

7. On page 6 (lines: 136-138) the author mentioned: “For all question items of PSQ-18, responses were structured across five types; ’Strongly Agree’, ‘Agree’, ‘Uncertain’, ‘Disagree’ and ‘Strongly Disagree’ with values ranging from 1 to 5 (See Table B in S1 File).” According to Table B in S1 File, it should state that for negative statements the scale value was reversed.

**Response:** Thank you for your helpful observation. As suggested, we have updated the manuscript to clarify that for question items that were negatively worded, the scale values were reversed following the scoring guidelines of the PSQ-18 in **Page 7, Line 156-57.**
This revision has been made in the Methods section, and Table B in the S1 File already reflects the correct scoring structure.

8. Existing literature suggests that the Rohingya people are often reluctant to talk with unknown people. How did the researchers handle the issue during data collection? What is the role the Majhi played in this regard?

**Response:** Thank you for this important observation. We have addressed these issues in **Page 6-7, Lines 142-147**. We recruited data collectors with substantial prior experience working in the Rohingya camps and fluency in the Chattogram dialect, closely resembling the Rohingya language. In addition, community leaders (Majhi) related with the study sites were informed about the study’s background, objectives, data collection procedures, voluntary nature of participation, confidentiality of responses and the expected outcomes, which helped build trust and support within the community.

9. Please rephrase the variable “type of illness” to “perception of the type of illness” since the study did not use medical reports to verify whether conditions were acute or chronic.

**Response:** Thank you for this insightful suggestion. We agree with your observation and have rephrased the variable from “type of illness” to **“perception of the type of illness”** in the narrative and tables generated as this classification was based on patient self-report, not clinical verification.

10. As the study participants are living in a humanitarian condition and refugee settings, it is expected that there is no significant variability in some of their characteristics. It is also difficult to change their socio-economic status in this current context. Accordingly, the authors could include some interventional variables such as health awareness, NGOs/INGOs intervention, or access to health information. I think it could be mentioned as one of the study’s limitations.

**Response:** Thank you for this valuable suggestion and we absolutely agree with you. We incorporated your advice as one of our limitations in **Page 20, Lines 352-355**. However, these variables were beyond the scope of our current study.

11. The study should perform a multivariate analysis like regression to identify significant predictors since bivariate analysis alone may not sufficiently report influential factors.

**Response:** Thank you for your thoughtful recommendation. Accordingly, we have performed **multivariate linear regression analysis** to identify independent predictors of patient satisfaction across its various dimensions. The results have been presented in **Page 16, Table 5**. We have also mentioned in **Abstract section (Page 2, Line 41-42)**, elaborated in the **Results section (Page 16, Lines 265-270)** as well as in **Discussion section** (Page 18-19, Lines 317-333).

12. In the abstract and results section, correct the notation from p = <.001 to p < .001.

**Response:** Thank you for pointing this out. We have corrected the notation in both the abstract and results section in accordance with standard statistical reporting conventions.

13. The surprisingly high patient satisfaction levels reported in this study, despite the ongoing struggles of the Rohingya with healthcare, might be explained by their extreme deprivation in their home country concerning basic health needs.

**Response:** Thank you for the insightful comment. We have addressed the surprisingly high satisfaction levels in the **Discussion section (Page 17, Lines 275-282)**. This may be explained by the contrast with past deprivation in Myanmar, as well as the structured service delivery and strong coordination among humanitarian actors in the camps.

14. The discussion section should include a paragraph about the implications of the study findings.

**Response:** Thank you for this thoughtful suggestion. We have now added a dedicated paragraph in the Discussion section **(Page 20, Lines 356-362)** on practical implications of our findings. This paragraph highlights how key domains of patient satisfaction—such as interpersonal manner, communication, and time spent with doctors—can inform policy development, guide targeted interventions, and support effective resource allocation in humanitarian health settings.

**Reviewer 2:**

**Reviewer Comment:**
In the method section it is mentioned that five primary health care facilities were selected randomly as study centers situated across five different Rohingya camps of Bangladesh. However, it is not clear whether the facilities were purely government or government, and NGO supported or purely NGO supported. If the PHC facilities are supported by NGO who were the donor. This information is important to understand the quality of providers, facility readiness, and the monitoring and supportive supervision done by the donor or government etc. as these issues are related to the quality of care provided. Adding this information regarding PHC facilities will help to understand the quality of care at PHC facilities and will help to set some standards in the recommendations about interpersonal manners, accessibility and convenience. Though authors mentioned in the limitation that provision of emergency allowance/incentives for working in humanitarian settings, working environment inside Rohingya camps, availability of essential diagnostics and medicines for beneficiaries were not evaluated.

**Response:** Thank you for this thoughtful and important observation. We agree that clarifying the nature of the selected primary health care facilities adds critical context regarding the quality of service provision and helps interpret the satisfaction levels observed. In response, we have revised the manuscript in **Methodology section (Page 5, Lines 105-106).** The selected health facilities were operationalized by non-government organization with financial support from international and UN agencies. In addition, we have reflected this observation in the **Discussion section (Page 17, Lines 278-282)**, noting that the structured nature of the service delivery model—supported by national NGOs and humanitarian partners—may have contributed to the high levels of satisfaction reported in our study.

Finally, We strongly believe that the reviewers’ comments have helped us to improve the presentation, readability and technicalities of the manuscript. We thank you again for your valuable comments.

Thank you.
